# Pharynx gargle samples are suitable for SARS-CoV-2 diagnostic use and save personal protective equipment and swabs

**DOI:** 10.1017/ice.2020.229

**Published:** 2020-05-11

**Authors:** Monika Malecki, Jessica Lüsebrink, Stefanie Teves, Andreas F. Wendel

**Affiliations:** 1 Institute of Hygiene, Cologne Merheim Medical Centre, University Hospital of Witten/Herdecke, Cologne, Germany; 2 Institute of Pathology, Cologne Merheim Medical Centre, University Hospital of Witten/Herdecke, Cologne, Germany

*To the Editor*—First described in China in December 2019, novel coronavirus (SARS-CoV-2), which causes coronavirus disease 2019 (COVID-19), has spread globally. Europe is now an epicenter of the COVID-19 pandemic. To bring the epidemic under control, laboratory testing for SARS-CoV-2 is essential to diagnose and isolate infected people and subsequently trace their contacts. Public health agencies recommend rapid initial testing by polymerase chain reaction (PCR) from upper respiratory (nasopharyngeal or oropharyngeal) specimens in ambulatory patients.^1,2^ SARS-CoV-2 replicates in the throat and the lung, and throat samples have a sufficient sensitivity in the first episode of the disease.^3^ However, healthcare workers (HCWs) obtaining the swab must wear personal protective equipment (PPE set: respirator, eye protection, gloves and gowns) because coughing might be induced by triggering the gag reflex of the person to be sampled.

In this pandemic, a foreseeable shortage of PPE and an acute lack of flocked swabs occurred in our hospital. Hence, we decided to collect pharynx gargle samples as an upper respiratory tract specimen (also known as oral rinse or throat wash in the literature). Pharynx gargle specimens can be obtained without close contact between the patient and the healthcare worker. Furthermore, pharynx gargle samples are easy to collect and sample the same anatomic region as throat swabs. Pharynx gargle samples are also an established method for the molecular detection of common respiratory infections, as well as in children.^4,5^ However, to our knowledge, only a few studies have assessed this type of specimen for the diagnosis of viral respiratory diseases. Bennet et al^5,6^ demonstrated that gargle samples were more sensitive in the detection of viral respiratory pathogens, and some evidence shows that gargle samples are suitable for SARS-CoV-2 diagnosis.^6,7^ Saliva collected by gargling has already been investigated for determining the viral load of SARS-CoV-2.^8^ In addition, gargle samples were successfully used in the first SARS epidemic for RNA detection and antigen testing.^9,10^

In March 2020, during the preparations for the first wave of infections, we installed examination units for HCWs in all 3 hospitals of our institution. HCWs were asked to come to the desk if they showed respiratory symptoms or if they had unprotected contact to a COVID-19 patient or SARS-CoV-2–positive HCW. We established the following workflow: The HCW approaches the desk, where a Plexiglas pane has been installed, and keeps a distance of at least 1.5 m. When symptomatic, the HCW wears a face mask. If a test is deemed necessary, he or she is instructed to provide a pharynx gargle sample in an empty room nearby equipped with a test kit (specimen container, 10 mL normal saline). After sampling (gargling time, 10–30 s), the closed container is left in the room. The throat wash is quickly transferred to a biosafety 2 laboratory and is subjected to a reverse transcription PCR for SARS-CoV-2 detection (RealStar SARS-CoV-2 RT-PCR Kit, Altona Diagnostics, Germany). On a regular basis, windows are opened in the sampling room, and contact sites are disinfected after each visit. During the whole procedure, no additional special PPE or swabs are needed.

From mid-March until April 20, 924 HCWs were tested at least once, and 26 samples were positive (2.8 %). Due to the limited number of PCR reagents and swabs, we examined only a very limited number (n = 5) of paired specimens (throat swab and gargle sample taken within 24 hours) in our hospital. We have observed 1 discrepant result (ie, throat swab negative and gargle sample positive) so far. At the same time, we saved at least 225 PPE sets (conservative calculation of 3 sets per day and per hospital over a period of 25 work days) and 1,000 swabs.

Of course, this approach can only be used if the person being tested is able to gargle. Patients from whom a gargle sample cannot be obtained (eg, dysphagia, dementia or infants) should be swabbed. Gargle samples might only be manageable for laboratories if there are low numbers of specimens. The gargle sample container is bigger than a swab; thus, it might cause problems with packaging or take too much space in a safety cabinet. Finally, in some countries national guidelines do not allow gargle sampling.

In conclusion, self-collected gargle samples are easy to take, noninvasive, material saving, and safe for healthcare workers. Nevertheless, more preanalytic data and comparative studies are needed at different stages of COVID-19.
